# Management of hyperacusis in children – two case reports^☆^

**DOI:** 10.1016/j.bjorl.2016.02.001

**Published:** 2016-04-11

**Authors:** Tanit Ganz Sanchez, Isabella Marques Pereira

**Affiliations:** a Universidade de São Paulo (USP), Faculdade de Medicina, Departamento de Otorrinolaringologia, São Paulo, SP, Brazil; b Instituto Ganz Sanchez, São Paulo, SP, Brazil; c Universidade Federal de Minas Gerais (UFMG), Faculdade de Medicina, Belo Horizonte, MG, Brazil

## Introduction

Hyperacusis is known as an abnormal reduced tolerance to environmental sounds.^1^ Such patients describe that everyday sounds are too loud and cause them discomfort or even pain.

This medical condition may induce a strong impact on the quality of life because subjects usually avoid common situations, such as social, familiar or professional interactions, public transport and walking on the streets.

The diagnosis includes a history of intolerance to different types of sounds plus decreased thresholds obtained in the Loudness Discomfort Levels (LDL) or Uncomfortable Loudness Levels (ULL) measured at least to 500, 1000, 2000 and 4000 Hz.^1^, ^2^

Hyperacusis is underestimated among children and adolescents, but it can be recognized when they react covering their ears, crying, screaming or leaving the place when they are exposed to television, games, conversation, telephone calls, home appliances (vacuum cleaning, washing machines, blender and others), car ride, and even going to school.^2^

In children, hyperacusis has been associated with tinnitus.^3^ About 86% of hyperacusis patients perceive tinnitus and 27–40% of tinnitus patients report hyperacusis.^4^

Medication is rarely described for treating hyperacusis, even for adults but especially for children/teenagers, because it would have to combine safety with efficacy. There is no drug with such proved qualities so far. However, Pharmacology has many examples of drugs that are used for further indications than those which they were developed for. Thus, we considered some characteristics of the *Ginkgo biloba* leaf extract, which is an over-the-counter herbal medicine widely prescribed for managing memory and concentration problems in different ages, depression, anxiety, dizziness, and tinnitus.^5^ When it was targeted for managing tinnitus, a commonly associated symptom, *G. biloba* proved to be safe,^6^ although its efficacy was controversial.^7^

Our aim is to describe the successful treatment of two male children with troublesome hyperacusis through a combination of medication plus sound enrichment.

## Case reports

### Case 1

PFB, a 12 year-old male, came for the first visit in February 2013, accompanied by his mother. He complained of sound intolerance to the voices of family, friends and teachers, radio, TV and traffic. At school, he had limitations in class, especially during physical education, when sports activities were practiced indoors. He started to use ear protection all day long and to spend the school recess in the silence of the library. Two months before the first visit, he complained about a constant high-frequency bilateral tinnitus, which made the family search for medical help.

Ear, nose and throat examination was normal. The initial audiological battery included hearing thresholds from 250 to 16,000 Hz, Loudness Discomfort Levels (LDL) and tinnitus pitch and loudness matching (Table 1, Table 2, Table 3).

**Table 1:** Hearing thresholds from 0.25 to 16 kHz in both ears in the 4 exams performed between March 2013 and February 2014.

	0.25	0.5	1	2	3	4	6	8	9	10	11.2	12.5	14	16
*Right hearing thresholds (kHz)*														
dBHL in March 2013	5	10	5	10	5	5	15	10	10	10	5	0	0	0
dBHL in May 2013	5	5	0	5	10	5	10	10	10	5	5	5	0	0
dBHL in August 2013	10	5	5	10	5	5	15	10	5	10	5	5	0	0
dBHL in February 2014	5	10	5	5	5	10	10	5	5	5	5	0	0	0

*Left hearing thresholds (kHz)*														
dBHL in March 2013	15	10	10	15	15	15	20	10	5	0	0	−5	0	0
dBHL in May 2013	10	5	5	10	15	15	15	10	5	5	5	0	0	0
dBHL in August 2013	10	10	5	10	5	10	10	5	5	5	5	0	0	0
dBHL in February 2014	15	10	10	15	10	15	15	5	5	0	0	−5	5	0

kHz, kilohertz; dBHL, decibel Hearing Level.

**Table 2:** Loudness Discomfort Levels obtained from 0.5 to 4 kHz in both ears in the 4 exams performed between March 2013 and February 2014.

	0.5	1	2	4
*Right LDL (kHz)*				
dBHL in March 2013	65	60	50	40
dBHL in May 2013	65	60	60	55
dBHL in August 2013	65	65	60	55
dBHL in February 2014	65	60	60	65

*Left LDL (kHz)*				
dBHL in March 2013	50	50	50	45
dBHL in May 2013	55	55	55	60
dBHL in August 2013	65	65	60	55
dBHL in February 2014	65	70	65	60

LDL, Loudness Discomfort Levels; kHz, kilohertz; dBHL, decibel Hearing Level.

**Table 3:** Tinnitus pitch and loudness matching obtained in the 4 exams performed between March 2013 and February 2014.

	Tinnitus pitch (kHz)		Tinnitus loudness dBSL		Minimal Masking Level dBSL	
	Right	Left	Right	Left	Right	Left
March 2013	12.5	12.5	18	16	24	23
May 2013	12.5	12.5	7	8	15	15
August 2013	12.5	12.5	2	4	6	8
February 2014	11.2	11.2	3	4	14	8

kHz, kilohertz; dBSL, decibel Sensation Level.

Treatment was composed by:

- Counseling using an easy language, including the definition of hyperacusis and tinnitus, their possible etiologies and association, as well as the need to decrease the constant use of ear protection, which is likely to worsen the sound intolerance.
- Use of environmental low level sound therapy for 2–3 h during the day or night. However, PFB disagreed because he was afraid of withdrawing ear protection.
- Because of this resistance, we prescribed *Gingko biloba* extract 80 mg twice a day for 2 months (24 mg of glicosides Ginkgo-flavonoids and 6 mg of terpene lactones), based on previous studies^7^, ^8^ that showed its safety when used for vertigo in children and for tinnitus in adults (no study about its use for tinnitus in children or in hyperacusis was found). The concomitant use of environmental sound, as mentioned above, was reinforced.

In May 2013, PFB came again. He did not adhere to using environmental sound. He noticed gradual improvement of both tinnitus and hyperacusis with the medication. The second audiological battery is described in Table 1, Table 2, Table 3. Based on his clinical and audiological improvement, we suggested to keep the medication for a further three months.

In August 2013, he reported greater improvement and eventually decreased the use of ear protection. He stopped going to the library at school when his colleagues were going to the recess. Tinnitus was barely perceived for a few minutes before going to sleep. The third audiological battery is described in Table 1, Table 2, Table 3.

Because of his extra clinical and audiological improvement, as well as his mother's satisfaction with his behavior at home and school, we withdrew the medication. After six months, PFB came back for the fourth audiological battery in February 2014, which proved that his improvement in both tinnitus and hyperacusis was kept stable with no further treatment (Table 1, Table 2, Table 3).

### Case 2

BGA, an 11 year-old male, came for the first visit in November 2013 accompanied by his parents. He has always complained of sound intolerance to many sounds, especially for the last 8 months. He was continuously using ear protection at school, street and home, so as to avoid being annoyed with the voices of friends, family, school colleagues and teachers, as well as the traffic jam and snack bars. He had no past or present history of tinnitus.

Ear, nose and throat examination was normal. His audiological exam showed normal bilateral hearing thresholds from 250 to 8000 Hz (Table 4), with LDL ranging from 80 to 90 dB in both ears (Table 5) and normal acoustic reflexes.

**Table 4:** Hearing thresholds from 0.25 to 8 kHz in both ears in the audiometry brought by the patient and performed in his own city.

Hearing thresholds (kHz)	0.25	0.5	1	2	3	4	6	8
Right ear, dBHL, Nov 2012	10	10	10	5	5	5	5	5
Left ear, dBHL, Nov 2012	20	20	10	5	5	5	5	15

**Table 5:** Loudness Discomfort Levels (LDL) from 0.5 to 4 kHz in both ears, brought by the patient and performed in his own city.

LDL (kHz)	0.5	1	2	4
Right ear, dBHL, Nov 2012	90	90	80	85
Left ear, dBHL, Nov 2012	90	85	85	80

Due to the previous success obtained with the treatment prescribed to the patient PFB, which will be discussed later, BGA was also advised to use *Gingko biloba* extract plus environmental sounds for 3 months. After 5 months (May 2014), parents reported that they noticed an improvement in his behavior when facing sounds and that he was rarely using ear protection. We recommended him to keep the environmental sounds at night and to decrease the *G. biloba* to one pill a day for another month.

After three months (August 2014), his mother reported that BGA was able to enjoy a friend's birthday party for the first time and also to celebrate his own birthday with friends and music. Both treatments were withdrawn.

## Discussion

Prevalence studies in children are rare and results varied widely from 2% to 42% with clinical complaint of hyperacusis, but just 3.2% had low Loudness Discomfort Levels (loudness hyperacusis).^3^, ^8^

Both cases described are pre-adolescent males suffering from hyperacusis. One also reported tinnitus, which is a common association.^3^ Within the auditory system, abnormally high neural gain may result in higher spontaneous and/or stimulus-evoked neural firing rates, resulting in tinnitus and/or sound intolerance.^9^ Our patient with tinnitus indeed had his LDL thresholds worse than the one without tinnitus.

A key point for the diagnosis is the measurement of ULL (Uncomfortable Loudness Levels) or LDL (Loudness Discomfort Levels), but the verbal instruction made by the audiologist is crucial to determine reliable values. For example, different results of ULL for asymptomatic listeners can be obtained when they point out they are “slightly uncomfortable” or “definitively uncomfortable” to loudness levels. In normal-listener young adults, the LDL varied between 86 and 98 dBHL from 250 to 8000 Hz.^2^ The following instruction was previously suggested and was followed in the present study: “You will hear sounds that will become louder. Please, press the button/raise your hand when the sound reaches an intensity that you no longer want to hear it, and the sound will stop immediately. We want to know which intensity provokes discomfort, and not if the sound is strong or weak. The sound can be strong and not provoke any hearing discomfort, for example. This test does not offer risk to your hearing even if you hear a sound in the maximum intensity of this equipment”.

Due to the heterogeneity of LDL obtained in normal listeners, the test should be carefully considered^2^; if the patient has the combination of clinical complaint about sound intolerance and discomfort thresholds lower than 95 dBHL, the diagnosis of hyperacusis would be adequate.

Tinnitus retraining therapy proposes counseling and sound therapy to manage hyperacusis and tinnitus. It was recommended for both patients, but only BGA performed it regularly together with the medication. So, its role in the success of the treatment of hyperacusis is not clear.

The constant use of ear protection is a common attempt to prevent further discomfort when facing unexpected sounds, especially when subjects are annoyed by many sounds or when they have to move around areas with varying noise levels.^10^ Our patients were using overprotection all day long, which can decrease the auditory input to central auditory pathways and induce extra hypersensitivity to sounds. Therefore, infants and parents should be counseled to keep the protection just when needed.

*G. biloba* extract seems to have influenced the improvement of hyperacusis in both cases. The rationale to use it in the first patient was the presence of short-term tinnitus, the lack of any evidence about using medication for tinnitus in children, and its safety in adults.^6^ In children or adolescents, it is used for attention-deficit hyperactivity disorder, dyslexia, and dizziness, among others.

In PFB's follow up, there was an improvement in his behavior and in his measurements of LDL and tinnitus loudness matching. So, the medication, although empirically tested, was able to partially improve tinnitus and hyperacusis. Thus, as a natural scientific step after having explained the previous experience to BGA's parents and having obtained their approval, we tried the same schedule for BGA when he complained of hyperacusis – with no tinnitus. No side effects were reported, in agreement with the literature.

Medication is rarely described for treating hyperacusis because a consistent and scientifically proved combination of safety and efficacy has not yet been described. However, Pharmacology and the drug market have many examples of drugs that are used for further indications than those which they were developed to. Some stories started with incidental findings which, in case of being well proven, may allow relief for a greater population. Considering that: (A) *G. biloba* leaf extract is an over-the-counter herbal medicine widely prescribed for managing memory and concentration problems in different ages, depression, anxiety, dizziness, and tinnitus^5^; (B) when it was targeted for managing tinnitus, a commonly associated symptom, *G. biloba* proved to be safe^6^, ^7^; (C) the patient PFB had tinnitus together with hyperacusis and the degree of control that *G. biloba* promoted in both symptoms was surprising; (D) the authors of the present report routinely explain the advantages and disadvantages of treatments, so that patients and/or parents have a participative role in choosing the best option in each case; (E) BGA's parents decided to search for help out of their country due to the severity of his symptoms and the lack of help in previous attempts; moreover, they were adequately oriented about the empiric effects of *G. biloba* in the first patient and about the lack of other scientific evidences to support it; with such information, their permission was given to try to use the same medication, this time in order to control just the hyperacusis. Despite the inherent risk taken by both sides, the success in controlling hyperacusis and returning quality of life to BGA was even greater, as the mother's emails have shown.

Medication was seldom reported for hyperacusis, even in adults: alprazolam (a short-acting anxiolytic), carbamazepine (an anticonvulsant and mood-stabilizing drug) or antidepressants (fluvoxamine and fluoxetine).^10^ Our reports in two pre-adolescent males do not intend to affirm that *Gingko biloba* is effective for managing hyperacusis and/or tinnitus, but rather to be a first step and allow further considerations for future controlled studies. If confirmed, this treatment option would likely increase the interest of Otolaryngologists in studying and treating this population.

## Conclusions

Isolated or in association with the environmental sounds, *G. biloba* extract contributed to dramatically improve the restrictive symptoms of sound intolerance in two pre-adolescent children.

## Conflicts of interest

The authors declare no conflicts of interest.
